# Calcium spikes, waves and oscillations in a large, patterned epithelial tissue

**DOI:** 10.1038/srep42786

**Published:** 2017-02-20

**Authors:** Ramya Balaji, Christina Bielmeier, Hartmann Harz, Jack Bates, Cornelia Stadler, Alexander Hildebrand, Anne-Kathrin Classen

**Affiliations:** 1 Albert-Ludwigs-University Freiburg, Center for Biological Systems Analysis, Habsburgerstrasse 49, 79104 Freiburg, Germany; 2 Ludwig-Maximilians-University Munich, Faculty of Biology, Grosshadernerstrasse 2-4, 82152 Planegg-Martinsried, Germany

## Abstract

While calcium signaling in excitable cells, such as muscle or neurons, is extensively characterized, calcium signaling in epithelial tissues is little understood. Specifically, the range of intercellular calcium signaling patterns elicited by tightly coupled epithelial cells and their function in the regulation of epithelial characteristics are little explored. We found that in *Drosophila* imaginal discs, a widely studied epithelial model organ, complex spatiotemporal calcium dynamics occur. We describe patterns that include intercellular waves traversing large tissue domains in striking oscillatory patterns as well as spikes confined to local domains of neighboring cells. The spatiotemporal characteristics of intercellular waves and oscillations arise as emergent properties of calcium mobilization within a sheet of gap-junction coupled cells and are influenced by cell size and environmental history. While the *in vivo* function of spikes, waves and oscillations requires further characterization, our genetic experiments suggest that core calcium signaling components guide actomyosin organization. Our study thus suggests a possible role for calcium signaling in epithelia but importantly, introduces a model epithelium enabling the dissection of cellular mechanisms supporting the initiation, transmission and regeneration of long-range intercellular calcium waves and the emergence of oscillations in a highly coupled multicellular sheet.

Elevation of cytoplasmic calcium is elicited by several signaling pathways and regulates multiple cellular functions^1^. In excitable cells, these functions include muscle contraction^2^,^3^ or neurotransmitter release^4^. In non-excitable cells, calcium regulates transcription^5^, cell cycle progression^6^ or apoptosis^7^. Calcium mediates these functions by calcium-sensing effectors that translate calcium signals with varying spatiotemporal dynamics into specific cellular responses^1^,^8^,^9^,^10^,^11^.

Spatiotemporal dynamics of calcium signals have been extensively studied in tissue culture. Signaling patterns observed at the single cell level include calcium spikes, intracellular waves and more complex oscillatory behaviors. Often, these phenomena depend on production of the second messenger inositol-1,4,5-trisphosplate (IP3), which binds to IP3 receptors (IP3R) and stimulates the release of calcium from intracellular stores, such as the ER (Fig. S1A)^1^,^12^. As both IP3 production and IP3 receptor opening are subject to positive and negative feedback regulation by calcium and IP3, calcium signaling can include signal amplification and refractory dynamics that give rise to intracellular calcium oscillations^9^,^10^,^13^. Multiple models of how IP3-dependent single cell oscillations arise have been proposed^9^,^13^ but under which circumstances oscillations may occur in whole tissues is little understood.

Importantly, calcium signals have been observed to spread between neighboring cells in a process described as intercellular calcium waves. Intercellular waves may facilitate transmission of local information from an initiating cell to a global field of cells and thus provide an appealing mechanism underlying coordination of cell behaviors. In non-excitable cells, intercellular waves are thought to primarily involve exchange of calcium or IP3 through gap junctions^10^,^14^,^15^. Alternatively, paracrine signaling mediated by the release of ATP into the extracellular space^10^ or opening of stretch-activated calcium channels between mechanically coupled cells^16^,^17^,^18^,^19^ have also been proposed to facilitate signal transmission. While most studies on intercellular signals have been performed on cultured cells, waves have also been reported to occur in organs, like the liver, brain and blood vessels^10^, or during early invertebrate and vertebrate embryogenesis^17^,^20^,^21^,^22^,^23^,^24^. While it is currently unclear how intercellular signaling dynamics relate to physiological tissue functions *in vivo*, these observations highlight the importance of investigating the little understood spatial dynamics of intercellular calcium signals within larger fields of cells.

We were particularly interested in understanding calcium dynamics in epithelial tissues. Epithelial cells organize into large sheets that line the surface of organs, such as the lung, colon or skin. This allows epithelia to act as barriers between exterior and interior environments and to fulfill crucial functions such as protection, secretion or absorption (Fig. S1B). The necessity to maintain tissue integrity at all times requires epithelial cells to establish highly coordinated behavior. Previous studies on calcium dynamics in cultured epithelial cells have specifically described calcium oscillations at single cell level that were not coordinated between adjacent cells, and discussed the emergence of short-range intercellular calcium waves^25^,^26^,^27^,^28^. More recent studies suggest that calcium signals may regulate epithelial cell behaviors by directly promoting cell shape changes^29^,^30^,^31^,^32^,^33^,^34^,^35^,^36^,^37^. For example, rapid changes in intracellular calcium levels were observed in cells surrounding a wound site in *Drosophila* epithelia^35^,^38^,^39^,^40^. These changes correlated with local waves of actomyosin flow and cell constriction, and contributed to the formation of an actomyosin cable that assisted in wound closure. Thus, as in migrating cells, where calcium has multifunctional roles in cytoskeleton redistribution and force generation^41^,^42^, calcium in epithelia may play a fundamental but little understood role in the relay of mechanical information and generation of mechanical response.

To provide a more comprehensive description of spatiotemporal calcium dynamics in epithelial tissues and to explore the ability of calcium to coordinate behavior between a larger number of cells, we chose to study *Drosophila* wing imaginal discs. They are a well-established epithelial model system that is experimentally and genetically tractable, and amenable to imaging approaches. Imaginal discs undergo extensive cell proliferation and patterning in larval stages and thereby give rise to progenitor organs of adult structures, such as wings and eyes. By the end of larval stages, the wing disc contains about 40,000 cells arranged in a sac-like structure (Fig. 1A–C). The ‘disc proper’, is composed of columnar cells, whereas the ‘peripodium’ contains squamous cells. The disc gives rise to the future wing blade (pouch), the hinge and the thoracic notum. Most of the disc presents as planar epithelium, whereas the hinge is characterized by the development of pronounced folds. By virtue of patterning, the disc is divided into lineage compartments of anterior, posterior, dorsal and ventral fates^43^. While most calcium signaling components are highly conserved in *Drosophila*^44^, to date only few studies investigate calcium signaling in this widely studied tissue^40^,^45^.

Here we describe the emergence of extremely complex spatiotemporal calcium dynamics that include calcium spikes, oscillations and intercellular waves in *Drosophila* imaginal discs and other fly organs. We specifically describe a spatiotemporal pattern encompassing an oscillating, regenerative calcium wave capable of traversing thousands of cells. Importantly, intercellular wave front propagation was highly coordinated between neighboring cells and spatial propagation paths replicated in a remarkably similar pattern with each oscillation. While the *in vivo* function of such oscillations, intercellular waves and calcium spikes is open to further investigation, genetic experiments suggested that calcium may guide patterning processes and implied a role in actomyosin regulation *in vivo*. Our observations thus provide an entry point into understanding a novel type of spatiotemporal calcium dynamics and present a highly genetically amendable model to dissect oscillations and intercellular wave front propagation in multicellular tissues.

## Results

### Calcium oscillations in cultured imaginal discs *ex situ*

To investigate what type of calcium signaling patterns can occur in developing epithelia, we visualized calcium in wing imaginal discs using the genetically encoded calcium sensor GCaMP. We specifically employed GCaMP5G, which has a dissociation constant (Kd) of 460 nM for calcium^46^ and is thus sensitive to physiologically relevant calcium concentrations. We ubiquitously expressed GCaMP5G using the GAL4/UAS system^47^ under the control of actin (act5C) or tubulin (tub84B) promoters (Fig. S1C,D). We dissected GCaMP-expressing discs from third instar larvae and mounted them for imaging by adapting recently published protocols for imaginal disc *ex situ* culture^48^,^49^,^50^.

**Figure 1:**
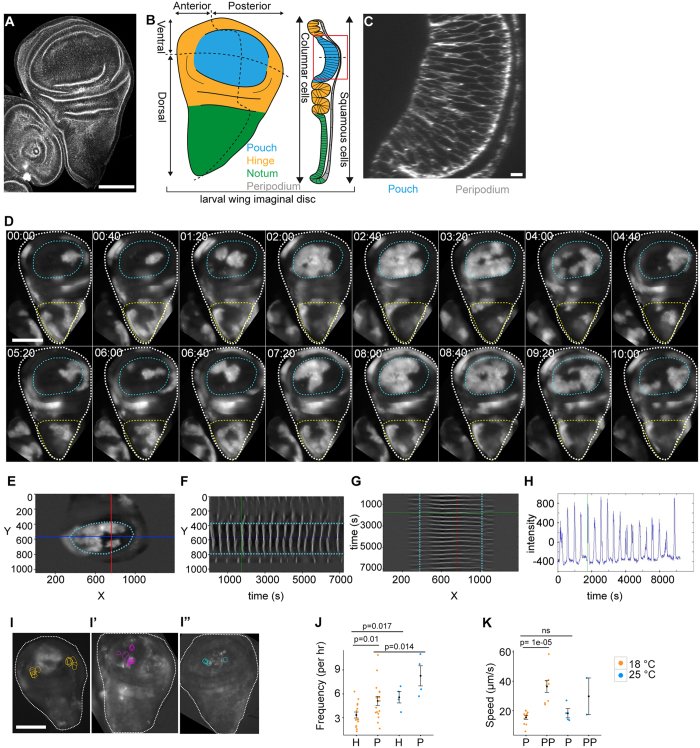
Oscillatory calcium waves in cultured imaginal discs *ex situ.* **(A)** Confocal section (XY) of a third instar wing imaginal disc labeled with Phalloidin. **(B)** Schematic representation of wing disc domains, compartments and cell shapes. **(C)** Cross-section (XZ) of wing pouch shown in (**A**). **(D)** Time series (mm:ss) of a GCaMP-expressing third instar wing disc (white outline), indicating the position of the pouch (cyan outline) and notum (yellow outline). GCaMP reporter activity visualizes oscillatory calcium waves. Time series (Movie S1) starts when oscillatory calcium waves have fully developed (see Fig. 4). (**E–G**) Wing pouch (cyan outline) of a GCaMP-expressing wing disc indicating position of line sections (red and blue) at which kymograph profiles in (**F**) and (**G**) were generated. YT (**F**) and TX (**G**) kymographs visualize GCaMP intensity in grey values. Intersection with pouch borders are tracked in cyan. Green line in (**F**) and (**G**) indicates time point shown in (**E**). Note different oscillation frequencies in the pouch and ventral/dorsal hinge domains and the inability of waves to traverse the pouch border (**F**). Note coupling of wave front propagation between pouch and anterior/posterior hinge domains in (**G**). **(H)** GCaMP-sensor activity at the intersection of the red and blue line segments shown in (**E**) over time. Green line indicates time point shown in (**E**). **(I–I”)** 3 examples of GCaMP-expressing wing discs for which position of recurring wave initiation points were mapped in the pouch. Each circle represents an initiation event. **(J)** Oscillation frequency in hinge (**H**) and pouch(**P**) domains. n = 17 wing discs at rearing temperature of 18 °C (orange) and n = 4 discs at rearing temperature of 25 °C (blue). Imaging was performed at 23 °C. **(K)** Speed of wave front propagation in the pouch (**P**) and peripodium (PP). Larvae were reared at 18 °C (orange, n = 11 pouches, n = 8 peripodia) or at 25 °C (blue, n = 4 pouches, n = 2 peripodia). Imaging was performed at of 23 °C. Graphs display Mean ± SEM, p-values were determined using a one-tailed Mann-Whitney test. Scale bars represent 100 μm in (**A,D** and **I–I”**) and 5 μm in (**C**).

Strikingly, we observed highly repetitive patterns of GCaMP-activity that were remarkably coordinated between several thousand neighboring cells (Fig. 1D). Domains of high GCaMP-activity shifted steadily across the tissue into adjoining low activity domains, thereby giving rise to the impression of wave-like calcium signals traversing the disc. These signals initiated in small sub-regions (Fig. 1D, Movie S1 t = 0:00–1:20), from where they travelled radially outwards to cover large tissue domains. As waves travelled outward, a decrease in calcium could be observed along the trailing wave edge, which occurred in a less coordinated fashion than expansion of high intensity signals at the wave front (Fig. 1D, Movie S1 t = 3:20–5:20). Upon reaching maximum extension, the wave front signal collapsed and decreasing calcium levels progressively spread to cover the entire area through which the wave had travelled (Fig. 1D, Movie S1 t = 2:40-6:00). After a delay, a new wave with similar spatiotemporal dynamics initiated in the sub-region where the previous wave had set in (Fig. 1D, Movie S1 t = 6:40-8:00). Because these signals reoccurred in a strikingly rhythmic pattern, henceforth, we call them oscillatory calcium waves.

To understand if the ability to initiate and propagate oscillatory calcium waves is specific to imaginal disc or if they also occur in other fly epithelial tissues, we performed *ex situ* imaging of follicle epithelia enclosing a growing egg chamber under the same conditions (Movie S2). GCaMP-expressing follicle epithelia exhibited pronounced oscillatory calcium waves suggesting that wave initiation, long-range propagation, cell-cell coordination, as well as oscillatory dynamics may be a general feature of fly epithelia.

### Oscillatory calcium waves are characterized by stochastic and deterministic features

To better understand the complex spatiotemporal dynamics of oscillatory waves, we characterized regional preferences, initiation origins, initiation frequency and wave front speed in wing discs. We identified three general regions where calcium waves predominantly emerged. A primary region was located in the wing pouch; a second in the notum (Fig. 1D, Movie S1); and a third in the peripodium (Fig. S1F). Calcium waves originating in the pouch could travel to cover most of the pouch area (Fig. 1D) but remained confined to the pouch by boundaries delineating the dorsal and ventral hinge folds (Fig. 1E,F). However, waves spread into the hinge via anterior and posterior pouch domains that are devoid of folds (Fig. 1E,G). Calcium waves that originated in the notum travelled primarily towards the dorsal hinge but failed to enter the pouch (Fig. 1D, t = 04:00 to t = 09:20). A third wave-initiating region was located in the peripodial membrane (Fig. S1F, Movie S3). Here, waves predominantly originated near the junction between the notum and peripodium and swept ventrally across the peripodial layer. Combined, these observations suggest that specific local regions within discs exist, where calcium waves are initiated and propagated efficiently. Strikingly, these regions may be separated by boundaries coinciding with tissue folds (Fig. 1B). However, calcium waves originating in the pouch of early third instar wing discs still failed to traverse into the hinge, despite the fact that folds are just starting to form at this developmental stage (Movie S4). This suggests that certain properties of developmentally patterned domains may facilitate or restrict calcium wave spreading.

To investigate if calcium waves always originated within the same exact sub-region of a disc, we mapped wave origins in the pouch of GCaMP-expressing wing discs. We found that although waves in one disc often initiated near the same location (Fig. S1G–I’), the location of wave origins varied from disc to disc (Fig. 1I–I”). This observation implies that local origins of oscillations may be subject to random fluctuations.

To better describe the temporal dynamics of oscillations, we quantified the frequency at which calcium waves occurred and the speed of wave front propagation. We found that, on average, 5.1 large waves traversed the wing pouch per hour (Fig. 1H,J, Fig. S2A,A’). In contrast, only 3.3 waves could be observed in the hinge (Fig. 1J, Fig. S2A). Because the hinge did not initiate waves itself, most waves observed here had spread anteriorly and posteriorly from the pouch. Thus the reduced frequency reflects the reduced likelihood of wave fronts to reach dorsal and ventral hinge positions far away from initiation points in the pouch.

Strikingly, we noticed a remarkable dependence of oscillation frequencies on the rearing temperature of larvae. In wing discs of third instar larvae reared at 25 °C instead of at 18 °C, the average oscillation frequency rose to 8.2 waves and 5.6 waves per hour in the pouch and hinge, respectively (Fig. 1J). Developmental age at imaging, wing disc size and cross-sectional areas of cells are very similar in discs reared at these two different temperatures (Fig. S2B–D). Therefore, these parameters cannot account for the observed differences. As imaging was always performed at 23 °C, the differences observed at different rearing temperatures suggest that oscillation frequencies may reflect a metabolic tissue state that is determined by a history of environmental influences.

In contrast, when we quantified the speed at which calcium waves traversed the tissue (Fig. S2E), we did not detect any influence of rearing temperature. In the pouch, calcium waves propagated on average at 15.4 μm/s or 18 μm/s, if larvae were raised at 18 °C or 25 °C, respectively (Fig. 1K). However, we found that wave fronts propagated at 36.4 μm/s or faster in the peripodial membrane (Fig. 1K). The difference in propagation speed within the pouch and the peripodium must thus be due to intrinsic differences between these two tissues. Pouch and peripodium cells differ dramatically in their cell area. While the columnar pouch cells cover an average apical area of 4.4 ± 0.2 μm^2^ (Fig. S2D), the squamous cells of the peripodial membrane cover 113.5 ± 3.4 μm^2^. The difference in cell areas alters the number of cell membranes, which may confer resistance to signal propagation, as the wave front traverses across the tissue. A similar effect of cell area on calcium signal propagation has been described to occur during local spreading of wound-induced calcium flashes^40^. This suggests that oscillatory calcium waves may be propagated by gap-junction dependent cell-cell coupling mechanisms rather than by paracrine signals whose propagation speed should not scale with cell size.

Combined these observations demonstrate that imaginal discs integrate stochastic properties (i.e. randomly positioned initiation origin, trailing edge fluctuations) and more deterministic, stable properties (i.e. history-dependent frequencies, cell-size-dependent propagation speed) to give rise to complex oscillatory behaviors.

### Calcium spikes, waves and oscillations occur in larval tissues *in vivo*

**Figure 2:**
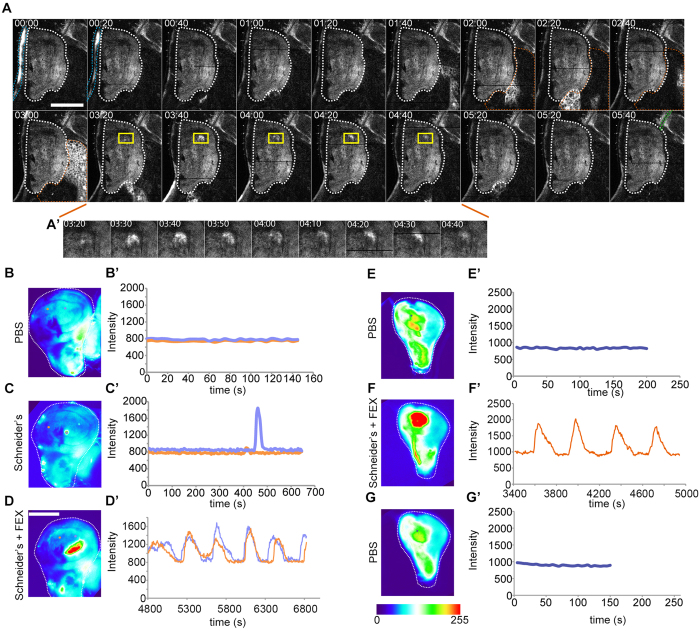
Calcium waves and spikes *in vivo* and *ex situ.* **(A–A’)** Time series (mm:ss) of GCaMP-expressing third instar larval tissues *in vivo* obtained by two-photon microscopy. GCaMP activity periodically oscillates in the larval epidermis (blue outline), fat body (orange outline) and trachea (green outline) (Movie S5). A calcium spike (yellow box) can be observed in the ventral pouch of a wing imaginal disc (t = 03:20 to 04:40), and is shown at higher magnification in (**A’**). **(B–D’)** Pseudo-colored movie stills (B-D) of GCaMP-activity in a wing disc imaged successively in PBS (**B**), Schneider’s medium (**C**) and Schneider’s medium supplemented with 5% fly extract (**D**). GCaMP-activity was traced over time (**B’–D’**) at position of orange and purple dots (**B–D**). Note that time axes are shown at different scales to better resolve changes in fluorescence intensities between short (PBS) and long (medium) treatments. Additional long-term experiments confirmed complete lack of calcium activity if discs were cultured only in PBS (Fig. S3A and Movie S6). 5, 8, and 22 independent experiments were performed for culture in PBS, Schneider’s medium and Schneider’s medium supplemented with 5% fly extract, respectively yielding the same result shown in (**B–D’**). **(E–G’)** Pseudo-colored movie stills (**E–G**) of GCaMP-activity in a wing imaginal disc imaged successively in PBS (E), Schneider’s medium with fly extract (**F**) and PBS (G). GCaMP-activity was traced over time (**E’–G’**) at position of orange and purple dots (**E–G**). Note that the graphs in D’ and F’ do not start at t = 0. This is because of a delay in the onset of oscillations (see Fig. 4). FEX = fly extract Scale bar represents 50 μm in (**A**) and 100 μm in B-G. Pseudo-colored scale bar represents GCaMP intensity values between 0 and 255.

To understand if oscillatory calcium waves also occur in a living organism, we performed whole-mount *in vivo* live imaging of third instar larvae that ubiquitously expressed GCaMP5G using 2-photon microscopy (Fig. 2A, Movie S5). To our surprise, we observed striking oscillatory waves traversing multiple larval organs *in vivo*. Specifically, we found repeating patterns of coordinated GCaMP-activity to spread across the tissues of the outer epidermal layer, the fat body or tracheal branches (Fig. 2A, Movie S5). This suggests that oscillatory calcium waves may be an inherent, though little understood, feature of epithelial and non-epithelial tissue function during larval development. However, in a limited number of whole-mount *in vivo* imaging experiments, we did not observe oscillatory calcium waves in wing discs of wandering third instar larvae. Instead, we observed spatially confined calcium spikes that travelled a short distance outward from the point of initiation (Fig. 2A’). Therefore, while many fly tissues appear to intrinsically generate oscillatory calcium waves, and imaginal discs are in principle capable of generating these patterns *ex situ*, our observations suggest that oscillatory calcium waves may not occur in imaginal discs under steady state conditions *in vivo*.

### Fly extract triggers calcium oscillations in imaginal disc culture

We therefore wanted to investigate if *ex situ* culture conditions induced calcium wave oscillations. To test if a specific component of the culturing medium composed of Schneider’s medium, insulin and fly extract is required, we cultured wing discs in PBS and Schneider’s medium alone or supplemented with insulin or fly extract. When discs were cultured in PBS, no evidence of elevated GCaMP-activity could be detected (Fig. S3A, Movie S6). When discs were cultured in Schneider’s medium with or without insulin, locally confined calcium spikes occurred (Fig. 2C,C’, Movie S7a). These spikes visualized as short-range signals that initiated at random locations in the disc and resembled calcium spikes observed *in vivo*. This suggests that calcium spikes may be an inherent feature of imaginal disc patterning. In contrast, when PBS or Schneider’s medium was supplemented with 5% fly extract, oscillatory calcium waves could be observed (Fig. 2D,D’, Movie S7b). When fly extract was exchanged with PBS oscillations ceased (Fig. 2E–G’). We therefore conclude that in *ex situ* disc culture, fly extract is specifically required to induce wave front propagation and oscillatory dynamics.

Consistent with the limited success *of ex situ* cultures^49^ we detected oscillations only during the first 2 hours of imaging, after which GCaMP-activity declined. As oscillatory waves were not observed *in vivo*, our data may imply that aberrant calcium influx could provide an explanation for why discs quickly deteriorate in fly-extract*-*supplemented *ex situ* culture. Aberrant signaling may interfere with developmental patterning, cell cycle progression^49^ and initiate pro-apoptotic pathways^7^. Thus, our observations point to a necessity to reevaluate currently published protocols for *ex situ* disc culture^49^,^50^,^51^.

### A calcium-mobilizing ligand is present in fly extract

To better characterize the nature of a calcium influx triggering signal in fly extract, we performed a series of simple experiments. Because fly extract is prepared from a largely female population of adult flies, we asked if extracts of female ovaries would be sufficient to induce oscillations. Indeed, ovary extracts induced mild oscillations (data not shown), as did extracts from whole larvae (Fig. S3B). In contrast, extracts from imaginal discs, larval brains or larval fat bodies did not elicit any response beyond that of spatially confined calcium spikes observed in Schneider’s medium alone (Fig. S3C). These experiments suggest that specific tissues at different developmental stages can contribute a calcium influx triggering signal to fly extracts.

To better characterize the potential biochemical nature of such a signal, we separated the fly extract using a centrifugal filter into two fractions containing molecules above and below 10 kD in size. The oscillation-inducing activity was retained by the fraction containing molecules larger than 10 kD, excluding nucleotides, small peptides or even steroid hormones as oscillation-inducing agents (Fig. S3D). In another series of experiments, we extended the heat-inactivation step during extract preparation from 5 min at 65 °C to 10 min at 100 °C and, strikingly, still observed calcium oscillations upon addition of the boiled extract to the culture medium (Fig. S3E). However, if the extract was treated for 1.5 h with Proteinase K, no calcium oscillations could be observed (Fig. S3F), even though oscillations could be induced in the same disc by addition of untreated fly extract afterwards (data not shown). Combined these experiments suggest that the functional agent is larger than 10 kD, extremely heat-stable but sensitive to protease digestion, indicating that the signal inducing calcium oscillations is either a protein or requires a proteinaceous component to induce calcium influx.

### Wave front propagation depends on IP3R, SOCE, ER-stored calcium and gap junction coupling

Consistent with the notion of a proteinaceous trigger, previous studies in non-excitable cells have often implicated extracellular ligand-triggered IP3-dependent calcium release from the ER^9^,^52^ in generating calcium oscillations. To provide evidence for a role of IP3 and ER-stored calcium in oscillatory waves, we pharmacologically interfered with IP3R, store-operated calcium entry (SOCE, Fig. S1A) and ER calcium-store functions.

**Figure 3:**
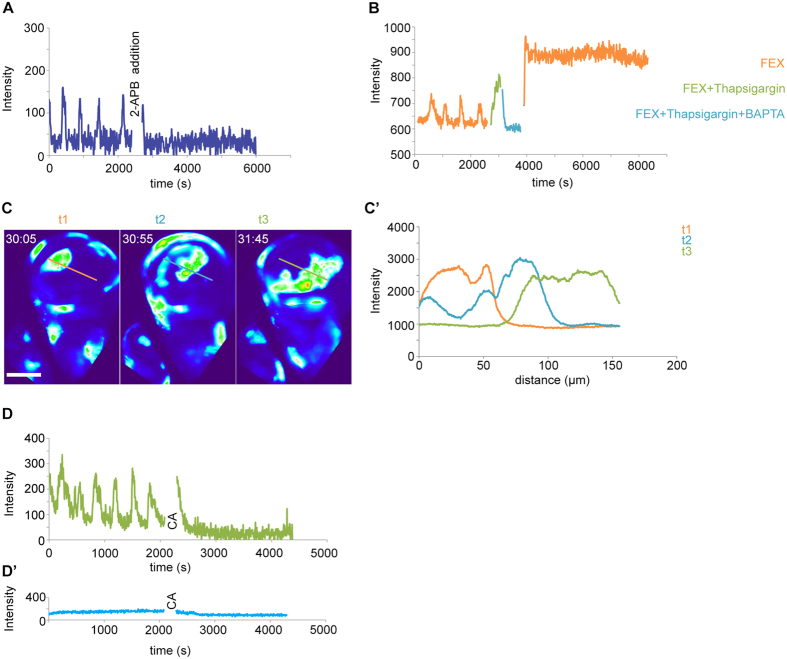
Oscillatory calcium waves depend IP3R, SOCE, ER-stored calcium and gap junction coupling. **(A)** GCaMP-intensity over time, pre- and post- 2-APB treatment of a disc cultured in Schneider’s medium with 5% fly extract. Graph represents trace at one representative position in a disc (n = 3/3 discs). **(B)** G-CaMP-intensity over time for a disc cultured in Schneider’s medium with 5% fly extract (FEX, orange), followed by treatment with Thapsigargin in FEX (green) and BAPTA in FEX (blue) and again in Schneider’s medium with 5% fly extract (FEX). Graph represents trace at one representative position in a disc (n = 3/3 discs). **(C,C’)** Line segment profiles of GCaMP-intensities measured at three subsequent time points t1 (orange), t2 (blue) and t3 (green) during expansion of a single wave front. Time given as mm:ss. **(D,D’)** GCaMP-intensity over time, pre- and post-Carbenoxolone treatment of disc cultured in Schneider’s medium with 5% fly extract (**D**) or PBS (**D’**). Graph represents trace at one representative position in a disc (n = 3/3 discs). CA - Carbenoxolone addition to the medium. Scale bar represents 100 μm.

The addition of the IP3R and SOCE inhibitor 2-APB^53^ to imaginal disc cultures completely blocked oscillatory calcium waves in the wing discs (Fig. 3A, Movie S8), implicating an IP3R and SOCE-dependent mechanism in oscillations. Strikingly, calcium spikes observed in discs cultured in Schneider’s medium are not sensitive to 2-APB treatment, suggesting that they arise by IP3R and SOCE-independent mechanisms (Fig. S4A,A’). Next, we tested the role of ER calcium stores in wave propagation by pharmacologically inhibiting SERCA pumps. SERCA pumps continuously refill ER calcium stores from cytosolic pools and can be irreversibly inhibited by Thapsigargin ref. 9. Upon treatment of oscillating imaginal discs with Thapsigargin, cytosolic calcium levels rose dramatically and coincided with cessation of detectable oscillatory dynamics (Fig. 3B, Fig. S4B, Movie S9). Upon addition of BAPTA, an extracellular chelator of calcium ions to Thapsigargin-treated discs, GCAMP-activity dropped to baseline levels (Fig. 3B, Fig. S4C, Movie S9) suggesting that cytosolic and ER calcium stores were depleted. After exchanging the culture medium containing BAPTA and Thapsigargin with culture medium containing fly extract, an increase in cytosolic GCAMP signals occurred but oscillations could not be observed (Fig. 3B, Movie S9). BAPTA and Thapsigargin treatment did not activate apoptosis or interfere with DNA replication up to 30 min post-treatment, suggesting that loss of oscillatory behavior was not due to acute loss of cell viability (Fig. S4D). This suggests that refilling of ER calcium stores is essential for oscillations to occur and that calcium fluxes at the plasma membrane or from other intracellular calcium stores, like mitochondria, are not sufficient to drive oscillatory dynamics.

Strikingly, after waves are initiated from a local region in the disc, wave front propagation is characterized by continuously high calcium activity. In fact, GCaMP signal amplitudes in the progressing wave front did not notably decrease as the wave propagated through tissue domains containing several thousand cells (Fig. 3C,C’). This suggests that wave front propagation is not maintained by passive diffusion of a cytoplasmic signal from an initiating point source. Instead, cell-autonomous regeneration of a transmittable signal appears to give rise to an active calcium wave. Given that calcium wave propagation often depends on cell-cell coupling by gap junction, we treated discs with Carbenoxolone, a chemical inhibitor of gap junction coupling^54^,^55^. Importantly, oscillations in cultured imaginal discs ceased immediately upon treatment with Carbenoxolone (Fig. 3D).

Combined, these data indicate that calcium waves in imaginal discs require gap junction mediated cell-cell coupling and is consistent with our observation that wave front propagation speed correlates with the number of cell junctions traversed. Our experiments furthermore implicate IP3R and SOCE-dependent release of calcium from ER stores in initiation and propagation of oscillatory waves. Importantly, oscillatory wave phenomena in non-excitable cells have been described to depend on IP3-mediated transmission of a regenerative calcium mobilizing signal^9^,^10^.

### Coordinated wave front propagation and oscillations are emergent properties of global calcium mobilization

**Figure 4:**
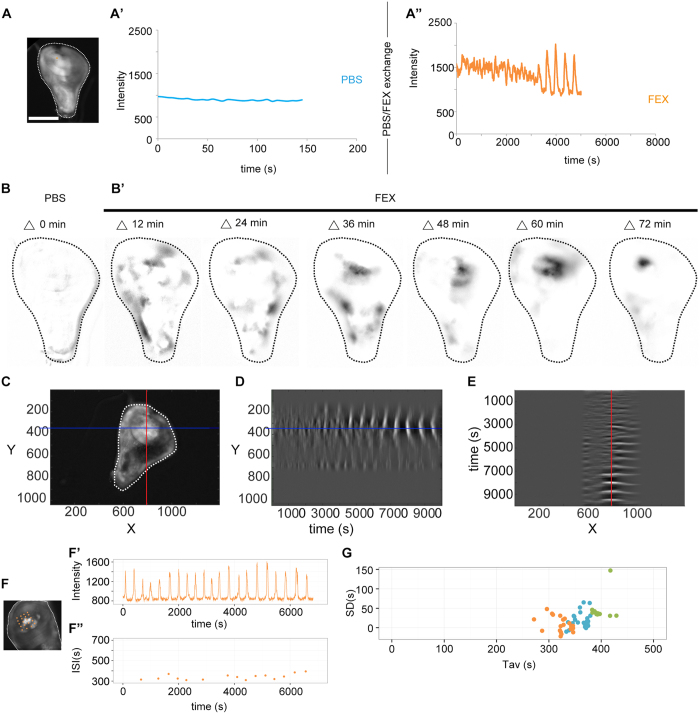
Oscillations are emergent properties of global calcium mobilization. **(A–A”)** Movie still of one representative wing disc (**A**) cultured in PBS (**A’**) and subsequently in Schneider’s medium supplemented with fly extract (**A”**). GCaMP-activity was recorded at positions indicated by an orange point (**A**) (see Fig. S5A–A” for another example). **(B–B’)** GCaMP-reporter activity in a disc cultured in PBS (**B**) and subsequently in Schneider’s medium supplemented with fly extract (**B’**) (same disc as shown in Fig. 2E–G’, Movie S10). Images were generated by subtracting the image at time point t from the image 2.5 min prior, where t = 2.5, 14.5, 26.5, 38.5, 50.5, 62.5, and 74.5 min in FIJI. Note that FIJI defines negative calculated values as 0. Images were subsequently inverted where pixel intensities of 0 = white and 255 = black. Note that number of moving calcium signals decreases with time and signal differences increase. **(C–E)** GCaMP-expressing wing disc (**C**) indicating position of line sections (red and blue) at which kymograph profiles in (**E**) and (**F**) were generated. YT (**D**) and TX (**E**) kymographs visualize GCaMP intensity in grey values. Note emergence of oscillatory calcium waves by enlargement of spatial domains covered by a single wave and increasing contrast between domains of high and low GCaMP activity. Disc analyzed is also shown in (**B**) and Movie S10. **(F–F”)** Representative GCaMP-expressing wing disc (**F**) with 20 grid points (orange) and intensity trace for one representative grid point (**F’**). Plot of corresponding inter-spike intervals (ISI) over time in F”. **(G)** Standard deviation (SD) of ISIs plotted against the average ISIs (Tav) per measured grid point in three independent wing discs (n = 25 (blue).15 (green) and 20 (orange) grid points for each respective disc). A linear regression analysis in R calculated R^2^ values of 0.1 (orange), 0.01 (blue), 0.03 (green) and the F-test for overall significance gave p-values of 01., 0.2, 0.2, respectively, demonstrating lack of linear correlation in these data sets. See Fig. S5B–B” for more details. FEX = fly extract Scale bar represents 100 μm.

To better understand how calcium oscillations arise as a consequence of fly extract stimulated mobilization of calcium from ER stores we more closely investigated the dynamics of oscillation onset. To avoid a delay in between mounting dissected discs in culture medium and the start of imaging, we dissected and mounted wing disc in PBS. Imaging them for several minutes confirmed that only background levels of GCaMP-activity were observed (Fig. 4A,A’, Fig. S5A,A’). However, exchanging PBS with 5% fly extract-containing medium caused an immediate and uniform increase in GCaMP-intensity across the entire disc (Fig. 4A”, Fig. S5A,A”, Movie S10). This demonstrates that the active agent in fly extract triggers an increase in cytosolic calcium in all cells of the disc, consistent with the expected uniform distribution of a ligand in the medium. Strikingly, we did not observe oscillatory waves at this point, suggesting that they only arise with time. Indeed, after 30–60 minutes, interspersed groups of neighboring cells exhibited a gradual reduction of GCaMP-activity to levels observed before the addition of fly extract to cultured discs (Fig. 4A”, Fig. S5A”, Movie S10 t = 25:00–50:00). After a delay, GCaMP-activity in these cells increased again to levels induced by the initial addition of fly extract (Fig. 4A”, Fig. S5A”, Movie S10 t = 50:00 onwards) and thereafter oscillated between both values. Importantly, a significant increase in cytosolic calcium is required for efficient induction of oscillations, as addition of just 1% of fly extract did elicit a rise in GCaMP-activity but failed to induce oscillations (not shown).

Another striking feature of oscillation onset was the gradual rise in the degree of coordination between increasingly larger domains of neighboring cells (Fig. 4B–E). When patches of low GCaMP-activity were first observed after the addition of fly extract, transitions between areas of high and low GCaMP-activity were gradual and several fluctuating domains could be observed within one disc (Fig. 4B–E, Movie S10, t = 00:00 to t = 40:00). However, 50 min into culture, sharp boundaries between high and low GCaMP-activity emerged and the number of fluctuating domains was strongly reduced (Fig. 4B–E, Movie S10 t = 50:00 onwards). To provide another measure for cell-cell coupling we quantified the variation in inter-spike intervals (ISIs) and analyzed the relationship between interval averages and standard deviations for different positions in oscillating discs (Fig. 4F–G, Fig. S5B–B”). Previous studies have shown that in uncoupled mammalian cells, the stochastic nature of calcium spikes renders this relationship linear^56^. In contrast, ISI analysis in oscillating discs revealed a lack of linear correlation between interval averages and standard deviations (Fig. 4G). This suggests that cooperativity between coupled cells underlies regular oscillations in imaginal discs and that the oscillator may have hard-wired properties constraining the possible range of oscillation frequencies.

Combined, our observations suggest that oscillations and wave front coordination are emergent properties of global calcium mobilization, of a machinery that works to maintain cytosolic calcium levels low and of a cell-cell coupling mechanism facilitating strong coordination between neighboring cells.

### A model for the emergence of calcium waves and oscillations

Based on these observations, we suggest a multi-step model for how oscillations arise (Fig. S6). First, all cells in the discs are stimulated by the uniform presence of a calcium-mobilizing agent potentially inducing production of IP3. Thereafter, reduction in intracellular calcium levels coincides with a refractory period in which cells cannot respond to the calcium-mobilizing agent anymore. As we implicate IP3R function in oscillations, calcium-induced inhibition of the IP3 receptor may induce refraction. Cell heterogeneity randomly positions cells that enter refraction earlier and therefore serve as symmetry breaking points that provide local vectors to wave front propagation (Fig. S6A). Thirdly, increasing coordination between neighboring cells into domains of high and low calcium signals is achieved by local cell-cell coupling which allows cell-cell transmission of a positive, regenerative signal. This transmittable signal needs to be able to be able to boost fly-extract-mediated stimulation of calcium mobilization to drive cells out refractory states. This would allow coordination of neighboring cells at heterogeneous positions in the refractory cycle. Lastly, calcium mobilization promotes signal regeneration to induce propagation of the wave front to the next cell (Fig. S6B). While the identity of a transmittable, regenerative signal requires additional experimental characterization, both IP3 (Fig. S6C,D,D’) and calcium (Fig. S6C,E,E’) could fit these requirements^10^.

### Calcium regulates patterning and actomyosin organization in the disc epithelium

While it is currently unclear if oscillatory calcium waves may also occur *in vivo*, calcium spikes observed in wing disc during *in vivo* imaging experiments appear to represent signaling events of yet uncharacterized function. To begin to understand if calcium signaling plays a role in imaginal discs, we genetically manipulated the function of several proteins required for calcium homeostasis using tools for RNAi-mediated knockdown or GAL4/UAS-mediated overexpression^44^. We targeted expression of these tools to the dorsal wing compartment by using the *apterousGAL4* driver (Fig. S1E). As previous reports link calcium to the regulation of the actin cytoskeleton in epithelial cells^33^,^34^,^35^,^36^,^38^,^39^,^40^, we first analyzed cellular architecture in discs by phalloidin and E-cadherin staining to visualize actin cytoskeleton and adhesion structures, and examined the appearance of adult structures developing from these discs.

**Figure 5:**
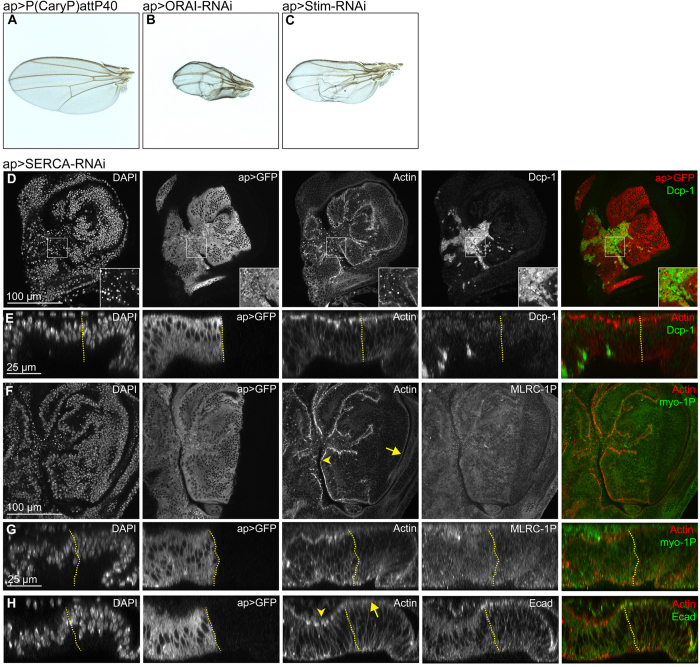
Calcium signaling affects patterning and actomyosin organization in the disc epithelium. **(A–C)** Control adult wings (**A**) and adult wings expressing ORAI-RNAi (**B**) and Stim-RNAi (**C**) under the control of apGAL4. Note the occurrence of blisters, vein thickening and the similarity of phenotypes in B and C. **(D–H)** Confocal xy sections (**D,F**) and xz cross-sections (**E,G,H**) of 3 wing discs expressing *SERCA-RNAi* under the control of *apGAL4. apGAL4* also drives expression of GFP (2^nd^ column) thereby positively marking the tissue where SERCA is downregulated. Non-GFP expressing cells represent internal wild type control tissue. All discs were stained for DAPI (1^st^ column) and Actin (3^rd^ column). One disc was also stained for the apoptotic marker Dcp-1 (**D,E**), another disc for the active form of the myosin regulatory light chain MRLC-1P (**F,G**) and yet another disc for the cell-cell adhesion protein E-cadherin (**H**) (4^th^ column). In overlays (5^th^ column), GFP or Actin are shown in red, the respective staining in green. Note the increase of apical actin intensities (3^rd^ column, compare arrowheads to arrows), increased levels of apical MRLC-1P (**F,G**), as well as the decrease of apical junctional E-cadherin staining intensities (**H**) in the GFP-positive dorsal compartment. Scale bars as indicated.

Manipulation of several components of the calcium homeostasis and calcium signaling machinery (i.e. SERCA, CamKII, Calmodulin or CanB2) caused nuclear fragmentation indicative of elevated levels of apoptosis in wing imaginal discs (Fig. 5D,E, Fig. S7A–C). Reduction of IP3R function altered cell heights in the dorsal pouch and reduced dorsal and, surprisingly, ventral hinge folds (Fig. S7D,E). While knock-down of components of the SOCE machinery (STIM, ORAI) caused blisters in adult wings (Fig. 5A–C), manipulating calcium-dependent signaling effectors (CanA, CanB, CamKI) produced defects in scutellar structure (Fig. S7F–J). Combined, these observations highlight the importance of calcium homeostasis and signaling for cell survival and implicate calcium in developmental patterning processes^45^.

Strikingly, however, we found that depletion of SERCA caused elevation of actin intensities in the apical cell cortex (Fig. 5F–H) and correlated with elevated levels of phosphorylated, and thus activated, non-muscle Myosin II regulatory light chain (MRLC-1P) (Fig. 5F,G). This coincided with loss of E-Cadherin (Ecad) from adherens junctions suggesting dramatic reorganization of actomyosin contractility and cell adhesion upon loss of SERCA function (Fig. 5H). Importantly, while SERCA depletion promoted apoptosis, changes to Actin, MRLC-1P and E-Cadherin levels were observed in tissue regions devoid of the apoptotic marker Dcp-1 (Fig. 5D,E). Combined these genetic experiments suggest that calcium signaling may be required for cortical actin homeostasis in imaginal discs and point to a potential regulation of cell-cell adhesion in epithelial cells.

## Discussion

Our work describes the striking phenomenon of oscillatory calcium waves in a large epithelial tissue. While future experiments need to dissect the potential occurrence of calcium oscillations in imaginal discs *in vivo*, our work highlights the principle ability of an epithelial sheet consisting of several 10,000 cells to give rise to incredibly complex spatio-temporal calcium dynamics. The experimentally accessible epithelial model of imaginal discs can thus be used to further dissect the currently little understood contribution of random and deterministic system properties to the emergence of highly coordinated intercellular calcium waves and oscillations.

We find that other fly epithelial tissues, such as trachea or epidermis, and non-epithelial organs, such as the fat body, exhibit remarkable oscillatory calcium waves *in vivo*. Recently, store-operated calcium entry in flies has been implicated as a key regulator of adiposity^57^, thereby providing a possible link to calcium oscillations in the fat body. Importantly, oscillation frequency in discs depended on the rearing temperature of larvae. Differences in metabolic rates at different temperatures may alter gene expression patterns and cellular calcium homeostasis, thereby altering the dynamics of wave initiation. While imaginal discs may not utilize metabolic encoding of oscillation frequency *in vivo*, this observation may have important implications for the cellular memory of tissues with metabolic functions, such as the fat body or adult gut stem cells^58^ and the mammalian liver^59^, in which calcium oscillations have also been observed.

The occurrence of calcium oscillations in the imaginal disc pouch and the notum strongly correlated with regions rich in Wingless signaling. In other systems, Wnt/Wingless signaling has been observed to activate calcium signals. For example, Wnt5a increases the rate of spontaneous calcium waves in Zebrafish embryos by activation of CamKII, which phosphorylates several regulators of calcium signaling^60^,^61^. Our observations raise the question if Wingless could pre-pattern calcium signaling dynamics in discs by similar mechanisms.

Clearly, the nature of the calcium-mobilizing ligand in fly extract requires additional characterization. However, we demonstrate that oscillations depend on ER calcium stores and cell-cell coupling by gap junctions and suggest that wave front propagation relies on transmission of a regenerative signal. In fact, a regenerative signal arising from IP3 and/or calcium transmitted through gap junctions would be consistent with the wave front propagation speed that we have measured^62^. As ryanodine receptors (RyR) and transient receptor potential (TRP) channels are essentially not expressed in imaginal discs^63^, signal transmission by RyR-dependent calcium-induced calcium release or by TRP-mediated calcium entry^1^,^3^,^53^ is probably not involved. Similarly, we found no evidence of signal transmission by ATP-mediated paracrine signaling or by stretch-activated calcium channels between mechanically coupled cells. Addition of ATP to cultured imaginal discs did not elicited any response (data not shown) likely because flies lack P2X receptors^64^,^65^. Similarly, mechanical wounding only initiates spatially and temporally restricted calcium waves^35^,^39^,^40^ and addition of the mechano-sensitive calcium channel inhibitor Gd^3+^ to oscillating disc cultures did not inhibit oscillatory calcium waves (Fig. S5C). However, treating discs with the Myosin II inhibitor Blebbistatin interrupted calcium oscillations (Fig. S5D). While this could be interpreted as inhibition of mechano-sensitive cell-cell coupling, inhibition of actomyosin-dependent endocytic trafficking of a receptor complex may similarly interfere with calcium mobilization.

Our work reveals several interesting features of oscillatory calcium waves in epithelial sheets. For example, a uniform increase of calcium across the tissue is required for a symmetry-breaking event to occur, where some cells more efficiently decrease cytosolic calcium levels than others and serve as symmetry breaking point for wave front initiation. In addition, a high level of calcium mobilization is required because reducing fly extract concentrations in the medium raised intracellular calcium levels but failed to induce oscillations. These observations echo dose-dependent effects described for agonist-induced IP3-dependent calcium oscillations in cells or dose-dependent responses of spiking to calcium availability in *Drosophila* embryos^13^,^20^. Importantly, we observed a pronounced latent period between fly extract stimulation and oscillation onset. Our optical resolution only allowed us to conclude on the average behavior of multiple neighboring cells. The lag phase may thus represent a period of increasing local coordination of single neighboring cells initially undergoing uncoordinated oscillations until they can be optically resolved.

Our genetic analysis of the cellular calcium machinery lacks the temporal resolution to provide direct evidence, at least for the role of calcium spikes *in vivo*. However, it allowed us to implicate calcium in cell viability, wing disc patterning, as well as actin cortex organization, E-cadherin localization and Myosin II activation. These observations are supported by a previously described requirement for calcium in the assembly and contraction of actomyosin networks in *Drosophila* follicle epithelia^37^, amnio serosa^32^, neuroepithelia^33^, or cultured mammalian epithelial cells^66^. Calcium-dependent actin-binding proteins, such as villin or gelsolin^67^,^68^, or calcium-dependent activation of Rho1 and Cdc42^36^ could mediate these processes.

In summary, our observations introduce a novel epithelial model system capable of complex spatiotemporal IP3R, SOCE, ER and gap junction-dependent calcium dynamics and implicate organization of the actin cortex and adhesion junctions as functional targets of calcium signaling. Future studies need to address how calcium oscillations and wave front propagation are coordinated by cell-cell coupling and global ligand stimulation, a phenomenon that at this scale has not been described before.

## Experimental Procedures

### Fly stocks and genetics

The following stocks were used: Act5C-GAL4/TM6b, Tub84B-GAL4/TM6b, UAS-GCaMP5G, ap-GAL4/CyO, ap-GAL4/CyO; UAS-mCD8 GFP/TM6c. For stocks used in Figs 5 and S7 please refer to Table 1. All stocks and experimental crosses were maintained on standard fly food at 18 °C or 25 °C.

### Imaginal disc culture

An *ex situ* disc culture protocol was adapted from^50^. Briefly, we supplemented Schneider’s medium with 5% fly extract and insulin to a final concentration of 3.1 μg/ml but omitted FBS. Extracts from larval and adult tissues were prepared as described in ref. 51. Relative extract concentrations are listed in Table 2. Proteinase K (SIGMA) treatment of fly extract was performed using 1/100 parts Proteinase K/extract volume for 1.5 h at 55 °C followed by heat inactivation at 90 °C. Fly extracts were separated into two fractions containing molecules above 10kD and below 10 kD in size using ultracentrifugal filter units (10,000NMWL, Amicon Millipore). Cultured imaginal discs were treated with Thapsigargin (Sigma) at 20 μM, BAPTA (ThermoFisher) at 10 mM, Carbenoxolone (Sigma) at 100 μM, 2-APB (2-aminoethoxydiphenyl borate) (Sigma) at 10 μM, Gadolinium(III) chloride hexahydrate (Sigma) at 100 μM and Blebbistatin (Sigma) at 100 μM final concentration in culture medium supplemented with adult fly extract. ATP treatment was performed in Schneider’s medium alone at a final concentration of 100 μM.

### Live imaging

Imaging chambers for *ex situ* GCaMP-activity recordings were set up as previously described^50^. A viscous medium containing 25 mg/ml methyl cellulose was used to seal the imaging chamber against leakage of the culture medium as described in ref. 48.

Imaging of GCaMP5G-sensor activity was performed on a setup developed in the BioImaging Center of the LMU Munich optimized for high acquisition speed and low phototoxicity. The set up was composed of a 470 nm LED as an excitation light source, a dual-band filter set optimized for DAPI and FITC fluorophores (DA/FI-A-000, Semrock, Rochester, New York, USA), a 20X 0.75 NA air objective (UPLSAPO, Olympus, Tokyo, Japan), achromatic tube lens (custom design), interline CCD camera (Clara, Andor, Belfast, UK) and software (Live Acquisition, version 2.6.0.1, FEI, Hillsboro, Oregon, USA). Time series (always denoted in mm:ss) were acquired at an exposure time of 70–100 ms and a frame rate of every 5 to 10 seconds for up to 2 hours at 23 °C. Imaging was paused when medium was exchanged to add or remove drugs.

To perform *in vivo* imaging of larval tissues, whole larvae were mounted as described in ref. 69. For two-photon measurements, a custom-build set-up of the BioImaging Center of the LMU Munich was used. A pulsed 900 nm laser was employed at 60 mV power (Mai Tai, Spectra Physics, Santa Clara, California, USA), 80 MHz pulse frequency, 10 μs/pixel laser dwell time, home-made scan head (digital scanner control, Smart Move, Munich, Germany), main beamsplitter LP 690, water immersion objective 25x NA 1.1 (CFI75 Apo LWD 25x W, Nikon, Tokio, Japan), photomultiplier (GaAsP photocathode, H7422P-40 MOD, Hamamatsu, Hamamatsu city, Japan), software (Colibri, freeware programmed in LavView, National Instruments, Austin, Texas, USA, https://github.com/C-Seebacher/colibri). Images were recorded at a frame rate of every 10 seconds.

### Immunohistochemistry and imaging

Imaginal discs were dissected and fixed in 4% formaldehyde/PBS for 18 min at 20 °C. Washing and blocking was performed in PBS + 0.1 Triton X-100 (PBT) and PBT + 5% normal goat serum, respectively. Discs were incubated with primary antibodies overnight at 4 °C: guinea pig anti-Spaghetti-squash 1 P (MRLC-1P) (1:400, gift from Robert Ward), rabbit anti-Cleaved *Drosophila* Dcp-1 (1:250) (Cell Signaling, 9578), mouse β-catenin (1:100, DSHB, N27A1) and rat anti-E-cadherin (1:100, DSHB, DCAD2). Discs were counterstained with DAPI (0.25 ng/μl, Sigma), Phalloidin (Alexa Fluor 488 and Alexa Fluor 647,1:100, Molecular Probes, or Phalloidin-TRITC, 1:400, Sigma). Secondary antibodies (coupled to Alexa Fluorophores, Molecular Probes) were incubated for 2 h at 20 °C. BrdU labeling on cultured discs was carried out as described^70^. Discs were mounted using Molecular Probes Antifade Reagents. Samples were imaged using a Leica TCS SP5 confocal microscope equipped with HCX PL APO Lambda Blue 20x (NA 0.7) and HCX PL APO Lambda Blue 63x (NA 1.4) lenses.

### Image quantification and statistical tests

Images and movies were analyzed in Fiji (ImageJ 1.48b), unless stated otherwise. Spatial profiles of GCaMP signals at different time points were obtained using the Plot Profile function. To generate intensity traces of GCaMP activity over time, the Z-axis profile function was employed on single pixel ROIs within the pouch of wing discs. Image calculator functions were used to obtain signal subtraction masks shown in Fig. 3. Image quantification methods to determine oscillation frequency and speed of wave front propagation are described in Fig. S2. For small data sets, a non-parametric, one-tailed Mann-Whitney test was performed (α = 0.05) (Fig. 1J,K). Datasets generated from the live imaging (x, y, intensity and t) could be navigated in a customized MATLAB program (MathWorks, Natick, Massachusetts, USA). The program allows to interactively select a point of interest in the wing disc (Fig. 1E) and displays the change of the intensity over time at this position in a line plot fluorescence emission intensity at the point x, y over time) (Fig. 1H) or display the intensity along the entire x or y axis over time (Fig. 1F–G). Image quantification methods to determine ISI averages and standard deviation (Fig. 4F–G) were performed as described in Fig. S5B–B” and ref. 56. Linear regression was determined in R using the linear least squares method. Data graphs were generated using Excel 2007 and R.

## Additional Information

**How to cite this article**: Balaji, R. *et al*. Calcium spikes, waves and oscillations in a large, patterned epithelial tissue. *Sci. Rep.* **7**, 42786; doi: 10.1038/srep42786 (2017).

**Publisher's note:** Springer Nature remains neutral with regard to jurisdictional claims in published maps and institutional affiliations.

## Supplementary Material

Supplementary Figures

Supplementary Movie S1

Supplementary Movie S2

Supplementary Movie S3

Supplementary Movie S4

Supplementary Movie S5

Supplementary Movie S6

Supplementary Movie S7a

Supplementary Movie S7b

Supplementary Movie S8

Supplementary Movie S9

Supplementary Movie S10

## Figures and Tables

**Table 1:** Genotypes presented in Figs 5 and S7.

Figure	Genotype	Bloomington stock crossed to *ap-GAL4, UAS-mCD8::GFP* driver
Figure 5		
A	*ap-Gal4/P(CaryP)attP40; UAS-mCD8::GFP*/+	BL36304 (control)
B	*ap-Gal4/UAS-Orai-RNAi; UAS-mCD8::GFP*/+	BL53333
C	*ap-Gal4/UAS-Stim-RNAi; UAS-mCD8::GFP*/+	BL41758
D–H	*ap-Gal4*/+*; UAS-SERCA-RNAi/UAS-mCD8::GFP*	BL44581
Figure S7		
A	*ap-Gal4*/+*; UAS-Cam-RNAi/UAS-mCD8::GFP*	BL34609
B	*ap-Gal4/*+*; UAS-CanB2-RNAi/UAS-mCD8::GFP*	BL38971
C	*ap-Gal4/UAS-CaMKII T287A; UAS-mCD8::GFP*/+	BL29663
E	*ap-Gal4*/+*; UAS-Itp-r83A-RNAi/UAS-mCD8::GFP*	BL25937
D,F	*ap-Gal4*/+*; P(CaryP)attP2/UAS-mCD8::GFP*	BL36303 (control)
G	*ap-Gal4*/+*; UAS-CanB-RNAi/UAS-mCD8::GFP*	BL27307
H	*ap-Gal4*/+*; UAS-CaMKI-RNAi/UAS-mCD8::GFP*	BL41900
I	*ap-Gal4/P(CaryP)attP40; UAS-mCD8::GFP*/+	BL36304 (control)
J	*ap-Gal4/UAS-CanA-14F-RNAi; UAS-mCD8::GFP*/+	BL38966

**Table 2:** Relative fly extract concentrations.

Extracts from	Number of animals*/*extract volume
Adult flies	200 flies in 1.5 ml
Total larvae	100 larvae in 1.5 ml
Larval brains and imaginal discs	20 larvae in 100 μl
Larval fat bodies	20 larvae in 100 μl
Adult ovaries	20 pairs in 100 μl
