# Supplementary material for: Calcium spikes, waves and oscillations in a large, patterned epithelial tissue
Source: Sci Rep. 2017 Feb 20;7:42786. doi: 10.1038/srep42786 (PMC5317010; doi:10.1038/srep42786)
Supplement: Supplementary Figures [file srep42786-s1.pdf]

Supplementary Information

Calcium spikes, waves and oscillations in a large, patterned epithelial tissue

Ramya Balaji [1,b], Christina Bielmeier [1,a], Hartmann Harz [a], Jack Bates [a], Cornelia Stadler [a], Alexander Hildebrand [a], Anne-Kathrin Classen [b],*

[1] shared first co-authors
* corresponding author

a Ludwig-Maximilians-University Munich, Faculty of Biology
Grosshadernerstrasse 2-4, 82152 Planegg-Martinsried, Germany

b Albert-Ludwigs-University Freiburg, Center for Biological Systems Analysis
Habsburgerstrasse 49, 79104 Freiburg, Germany

Supplementary movies

Note that stable patches of high and low GCaMP-intensity are due to variegated expression of the tubGAL4, actGAL4 and UAS transgenes in *Drosophila* tissues (see Fig. S1C,D and for an example [1]).

Time stamps in movies correspond to minutes:seconds (mm:ss).

The pixel resolution of all movies was reduced by a factor of 2 to provide the best compromise between data resolution and file size.

Movie S1
Calcium oscillations in wing imaginal disc *ex situ*, related to Figure 1D
Wing imaginal disc ubiquitously expressing GCaMP5G and cultured in Schneider's medium supplemented with 5% fly extract (see Experimental Procedures). Movie starts when oscillatory calcium waves are fully developed (see Figure 4).

Movie S2
Calcium oscillations in the follicle cell epithelium *ex situ*
A stage 7 egg chamber ubiquitously expressing GCaMP5G under the control of T155-GAL4 and cultured in Schneider's medium supplemented with 5% fly extract. A sheet of follicle cells encloses the growing germline consisting of large nurse cells and the oocyte. The focus of the movie shifts from a medial section through the egg chamber into the follicle epithelium by the end of the movie.

Movie S3
Calcium oscillations in the wing disc peripodium *ex situ*
Wing imaginal disc ubiquitously expressing GCaMP5G and cultured in Schneider's medium supplemented with 5% fly extract. The peripodium is located slightly out of focus but can be observed to generate sweeping GCaMP signals at t= 1:30 and t=13:50 onwards, which originate at the edge of the notum and travel across the wing disc.

Movie S4
Calcium waves initiating in the pouch do not traverse dorsal hinge folds in young third instar discs
Calcium waves traversing the pouch (t=2:10 to 4:40) do not enter the dorsal hinge, although a wave from the notum prior to this time point (t=0:00 to 1:35) enters the hinge domain.
Note that the out of focus wave observed at t= 3:40 and 4:45 is traversing the peripodium.

Movie S5
***In-vivo* imaging of calcium signals in whole mount larva**
A whole mount of a larva ubiquitously expressing GCaMP5G. For location of different tissues please refer to legend of Fig. 2A. In addition to the time series shown in Fig. 2A, a calcium wave can be seen in the trachea (t= 08:00 onwards).

Movie S6
GCaMP activity in wing disc cultured in PBS
A wing imaginal disc ubiquitously expressing GCaMP5G cultured for 2 hours in PBS.

Movies S7
Calcium oscillations in *ex situ* cultures are dependent on fly extract
A wing imaginal disc ubiquitously expressing GCaMP5G was sequentially cultured in PBS (not shown, see Movie S6), Schneider's medium (Movie S7a) and Schneider's medium supplemented with 5% fly extract (Movie S7b).

Movie S7a
GCaMP activity in a wing disc cultured in Schneider's medium

Movie S7b
GCaMP activity in a wing disc cultured Schneider's medium supplemented with 5% fly extract

Movie S8
Calcium oscillations in *ex situ* cultures are dependent on IP3R and SOCE function
GCaMP5G activity in wing disc cultured in Schneider's medium with 5% fly extract and then in Schneider's medium with 5% fly extract supplemented with the chemical inhibitor 2-APB.

Movie S9
Calcium oscillations in *ex situ* cultures are dependent on ER calcium stores
GCaMP activity in wing disc cultured sequentially in (1) Schneider's medium with 5% fly extract, (2) Schneider's medium with 5% fly extract supplemented with the chemical inhibitor Thapsigargin, (3) then in Schneider's medium with 5% fly extract supplemented with the calcium chelator BAPTA and finally with (4) Schneider's medium with 5% fly extract.

Movie S10
Coordinated wave front propagation and oscillations are emergent properties of global calcium mobilization
Wing imaginal disc ubiquitously expressing GCaMP5G and cultured in Schneider's medium supplemented with 5% fly extract.

Supplementary figures

Figure S1
Calcium signaling and oscillations in wing discs
Related to Figure 1.

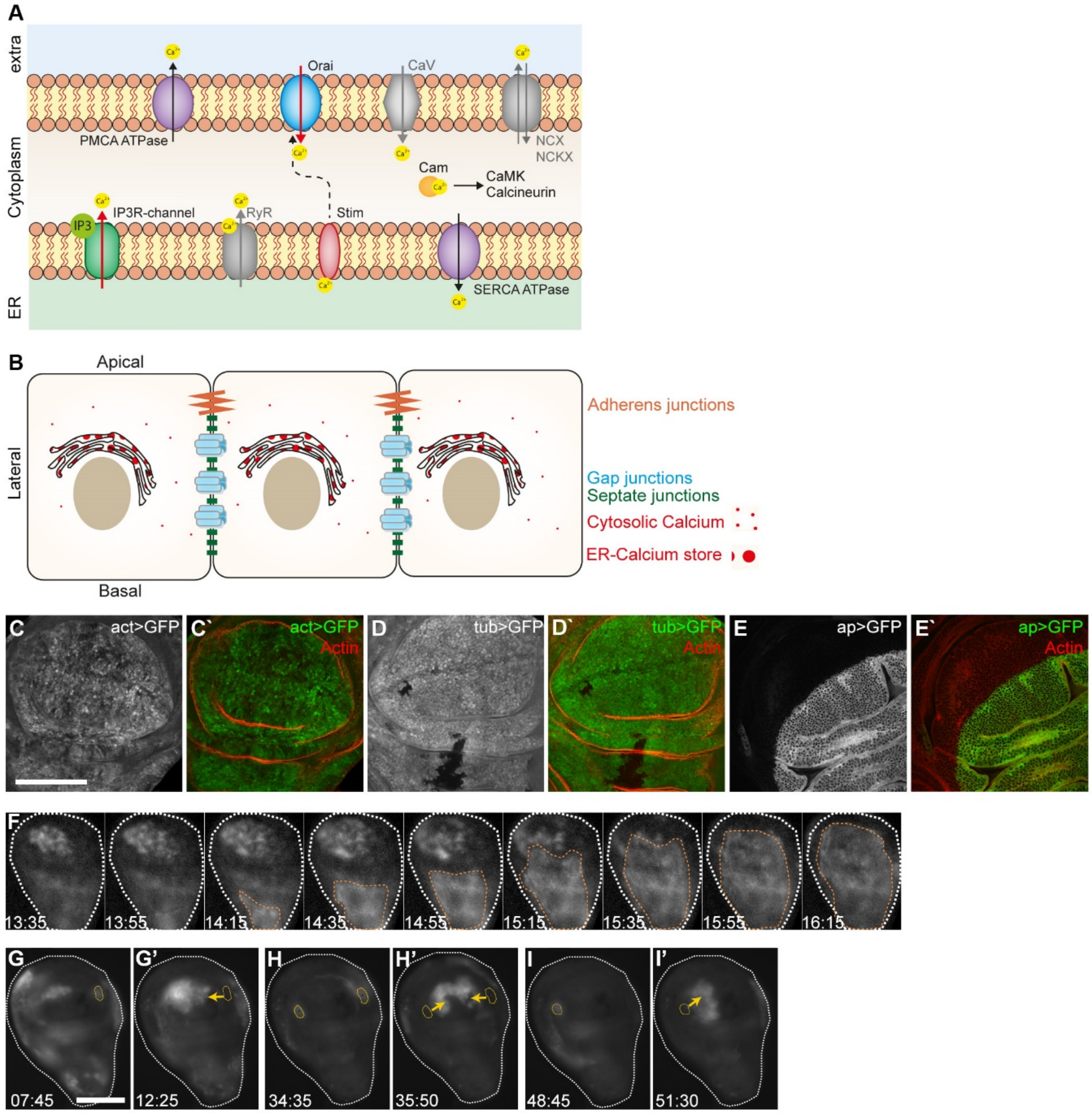

(A) Cytosolic calcium concentrations are tightly regulated by various components located either in the plasma membrane, the ER membrane or the cytoplasm. Membrane-resident ion pumps, such as the Sarco/endoplasmic reticulum calcium-ATPase (SERCA), the plasma membrane calcium ATPase (PMCA) or sodium/calcium exchangers (NCX and NCKX), are constantly removing calcium from the cytoplasm into the ER or into the extracellular space (extra), thereby decreasing cytosolic calcium concentrations. In addition, free cytosolic calcium ions are buffered by calcium-binding proteins including Calmodulin (Cam). In excitable cells, cytosolic calcium concentrations can increase by activation of voltage gated channels (CaV) upon depolarization of the plasma membrane and Ryanodine receptor (RyR) mediated release of calcium from the ER. In non-excitable cells, inositol-1,4,5-triphosphate receptor (IP3R) mediated release of

calcium from internal stores is the predominant source of cytosolic calcium. Restoration of ER calcium levels is mediated by SERCA, an ATPase on the ER membrane that continuously pumps cytosolic calcium back into the ER and by STIM-mediated activation of plasma membrane channels (CRAC, Orai), a process referred to as store-operated calcium entry (SOCE) [2,3]. Calmodulin mediates many calcium-dependent signaling processes by regulating various downstream effectors like Calcium/Calmodulin dependent protein kinases (CaMK) or phosphatases (Calcineurin) (see for example [3,4]). Note that calcium components shown in grey are not discussed in this study as they are not, or only at very low levels, expressed in imaginal discs [5].

(B) Schematic representation of *Drosophila* epithelia. Epithelial cells have a distinct apical-basal polarity and their lateral surfaces mediate cell-cell interactions. E-cadherin-containing adherens junction, as well as gap junction made from innexins (fly connexin) mediate mechanical and biochemical coupling of neighboring cells. Septate junction act as trans-epithelial barrier. The ER located near the nucleus acts as major intracellular store of calcium.

(C, D) Control experiment illustrating expression pattern of GAL4 drivers. Wing imaginal discs ubiquitously expressing UAS-GFP under the control of *act5C-GAL4* (C, green in C') or *tub84B-GAL4* (D, green in D') stained for actin (red in C', D'). Note that patches of very low GFP-expression visible in (D) are due to variegated expression of GAL4 and UAS transgenes in *Drosophila* tissues [1].

(E) Control experiment illustrating expression pattern of *ap-GAL4* driving UAS-CD8-GFP (E, green in E') in wing imaginal discs also stained for actin (red in E'). Expression is targeted to the dorsal wing disc compartment.

(F) Time series of calcium wave front propagation (orange outline) in the peripodium of a wing disc (white outline) cultured in Schneider's medium supplemented with fly extract. Time stamps correspond to minutes:seconds (mm:ss). See Supplementary Movie S3.

(G-I') Origin (yellow outlines) of three subsequent calcium waves in cultured wing discs were mapped. Image G, H, I show movie still at wave initiation, images G', H' and I' represent movie stills at maximum wave front expansion. Arrows indicate direction of calcium wave progression. Time stamps correspond to minutes:seconds (mm:ss).

Scale bars represents 100 μm.

Figure S2
Quantification work flows to determine oscillation frequency, wave front speed
Related to Figure 1

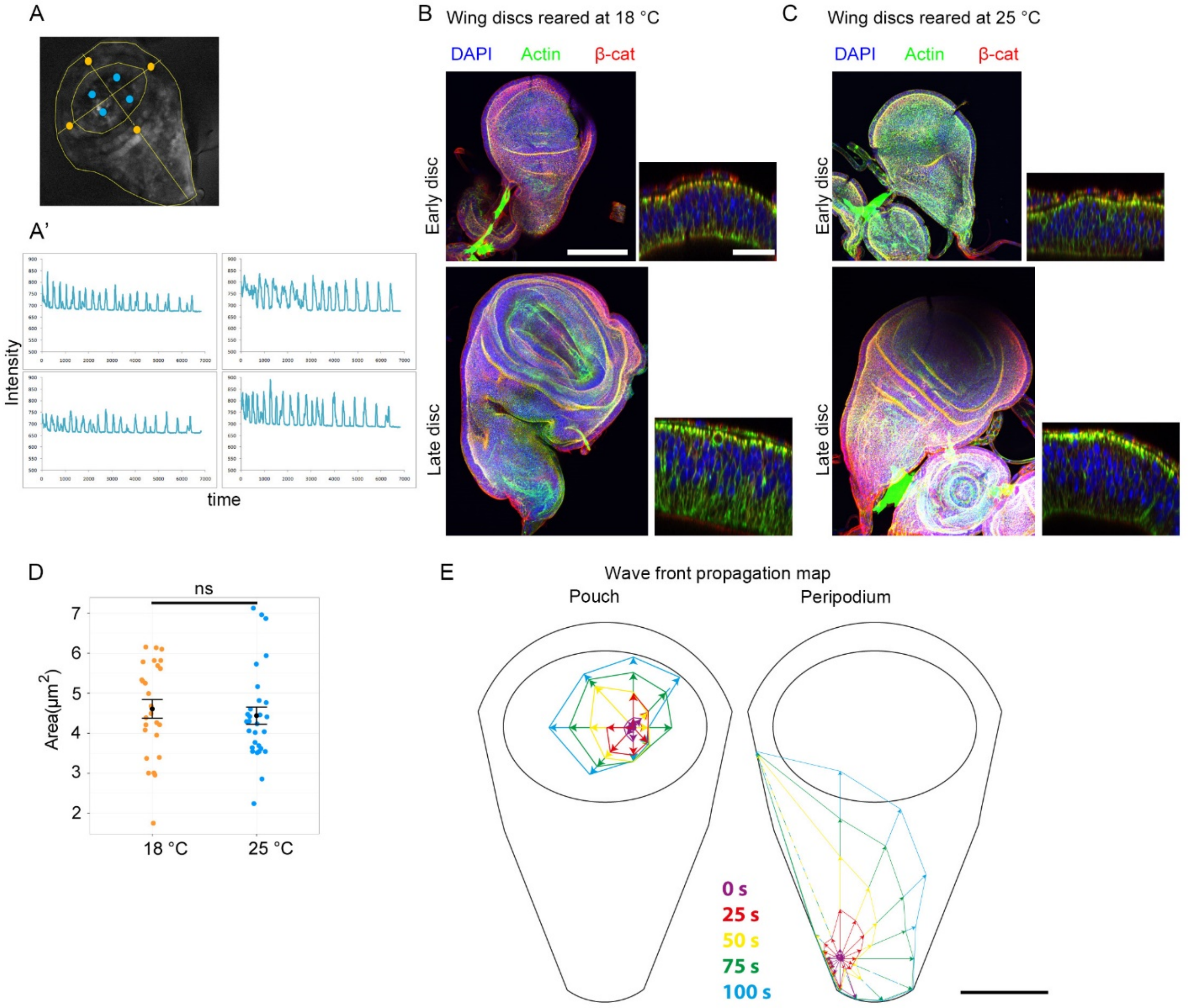

(A-A') To determine oscillation frequencies, GCaMP fluorescence intensity was traced (A') for 4 one-pixel points in the pouch (A, blue dots). Oscillation peaks are counted from these traces and averaged to calculate oscillation frequency in the pouch. Oscillation frequency in the hinge domain was calculated similarly at 4 selected one-pixel point positions (A, orange dots).
(B, C) Early and late third instar wing discs reared at 18°C (B) and at 25°C (C) labelled with DAPI (blue), Actin (green) and β-catenin (red). XZ cross-sections of each disc are shown adjacent to the disc and illustrate the morphological similarities in tissues raised at different temperatures. Scale bars on wing discs in (B and C) represent 100μm and in XZ cross-sections (B and C) represents 25μm.
(D) Mean apical cell areas for columnar cells of late third instar wing imaginal discs were 4.6 μm^2 (n= 26 cells) and 4.4 μm^2 (n=29 cells) if larvae were raise at 18°C or 25°C, respectively. Graph shows Mean ± SEM of areas.
(E) Wave front propagation for a single wave in the pouch and the peripodium. Wave fronts were visually mapped from their origin (purple, t=0 s) at 25 s intervals and were color-coded according

to the interval at which they were recorded. The distance the wave front travelled was mapped on a Cartesian coordinate system at 45° angles and every 25 s. The average speed of wave front propagation was calculated from all speeds recorded. These speed measurements assume that between time intervals, the speed of the wave front propagation is uniform. The scale bar represents 100 μm on a standard schematic disc.

Figure S3
Characterization of a calcium-mobilizing ligand in fly extract
Related to Figure 2.

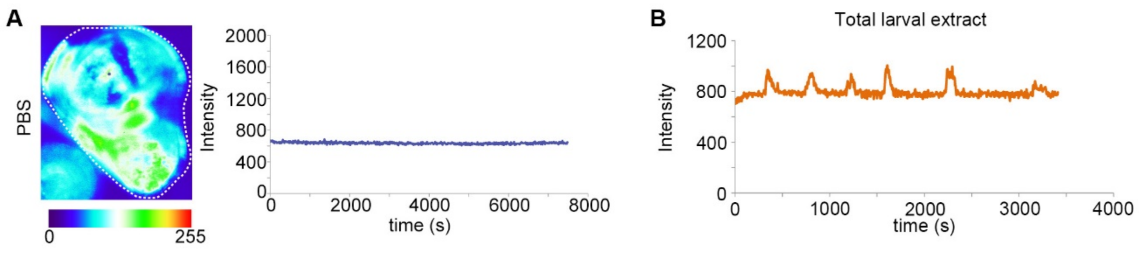

C

Tissue type	Calcium signal (Y/N) and description
LEX	Yes, but not as potent as FEX
Ovary	Yes, but not as potent as FEX
Fat body	No, just spikes
Larval brain and imaginal discs	No, just spikes

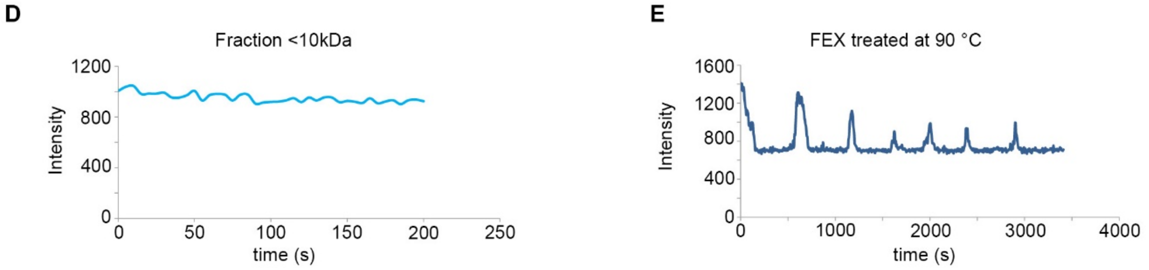

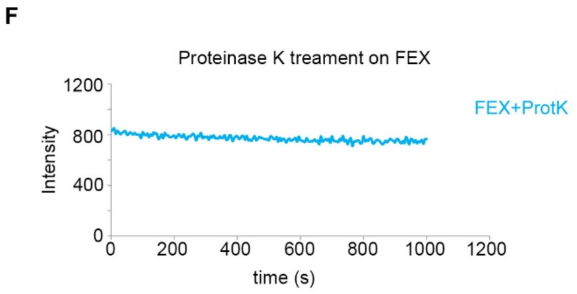

(A) GCaMP-intensity traced over time at a representative position (blue dot) in a wing disc cultured in PBS for over 2 hours.
(B) GCaMP-intensity traced over time at a representative position in a wing disc cultured in Schneider's medium supplemented with 5% extract from whole larvae.
(C) Table briefly summarizes the type of calcium signals observed upon supplementing the medium with different extracts. LEX = whole larval extract.
(D-F) GCaMP fluorescence intensity traced over time in wing discs cultured in Schneider's medium supplemented with 5% of a fly extract fraction containing molecules smaller than 10kDa in size (D), or supplemented with fly extract previously treated at 90°C (E), or supplemented with Proteinase K-treated fly extract (F).

Figure S4
Calcium waves depend on IP3R, SOCE and ER calcium stores
Related to Figure 3

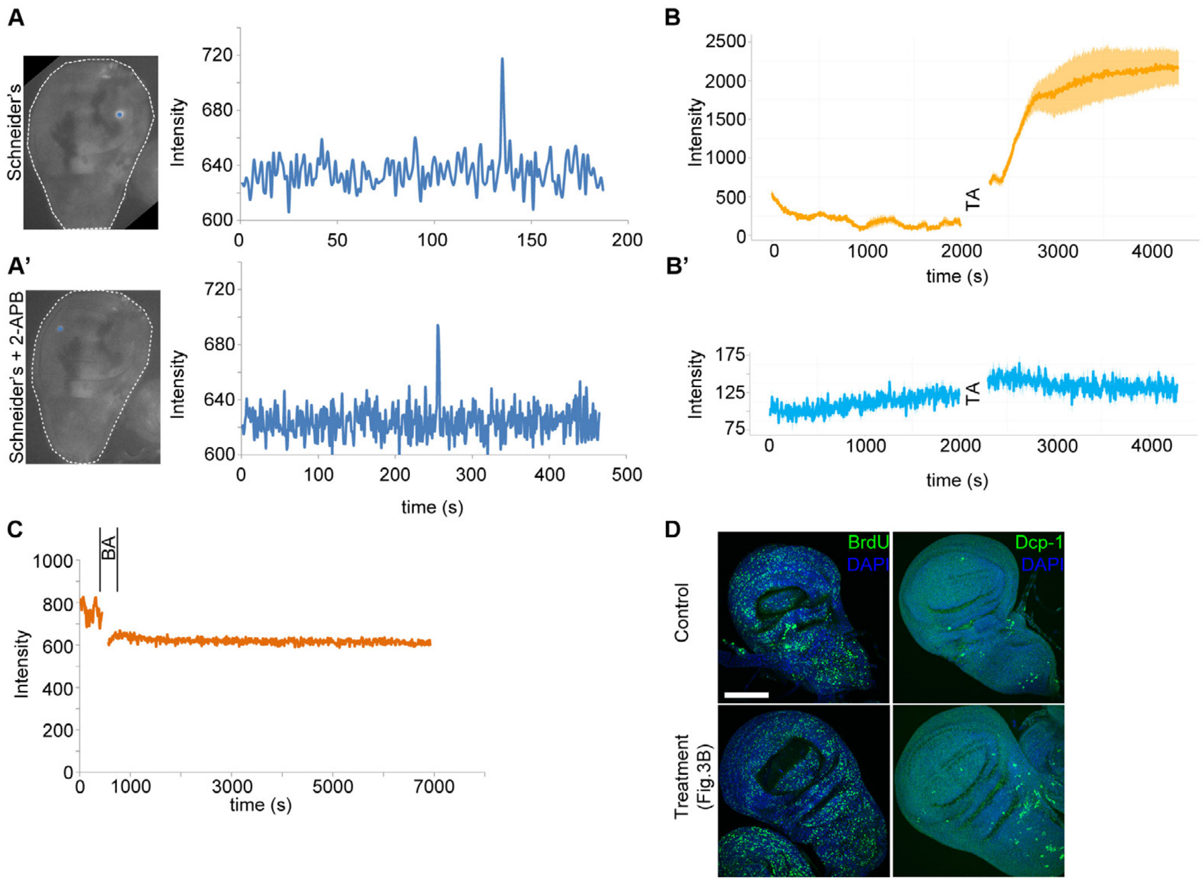

(A-A') GCaMP-intensity trace over time at a representative position (blue dot) in a wing disc first cultured in Schneider's medium (A) and then in Schneider's medium supplemented with 2-APB (A') (n=2/2 discs).
(B-B') GCaMP-intensity over time, pre- and post-Thapsigargin treatment of a disc cultured in Schneider's medium with 5% fly extract (B) or PBS (B'). Note that oscillation amplitudes prior to TA treatment are dampened because graphs represent the mean ± SEM of 4 data points in the disc. Three independent experiments were performed (n=3/3 discs). TA - Thapsigargin addition to the medium.
(C) GCaMP-intensity trace over time at a representative position in a wing disc cultured first in Schneider's medium supplemented with 5% fly extract, followed by addition of BAPTA (BA) (n=2/2).
(D) BrDU incorporation (BrDU, green) and activation of caspases (cleaved Dcp1, green) is not altered in cultured wing discs 30 min after being treated with Thapsigargin and BAPTA (treatment, see Fig. 3B) if compared to untreated control discs (control). Discs were counterstained with DAPI (blue).

Figure S5

Calcium oscillations are emergent properties of global calcium mobilization and do not depend on mechano-sensitive mechanisms
Related to Figure 4.

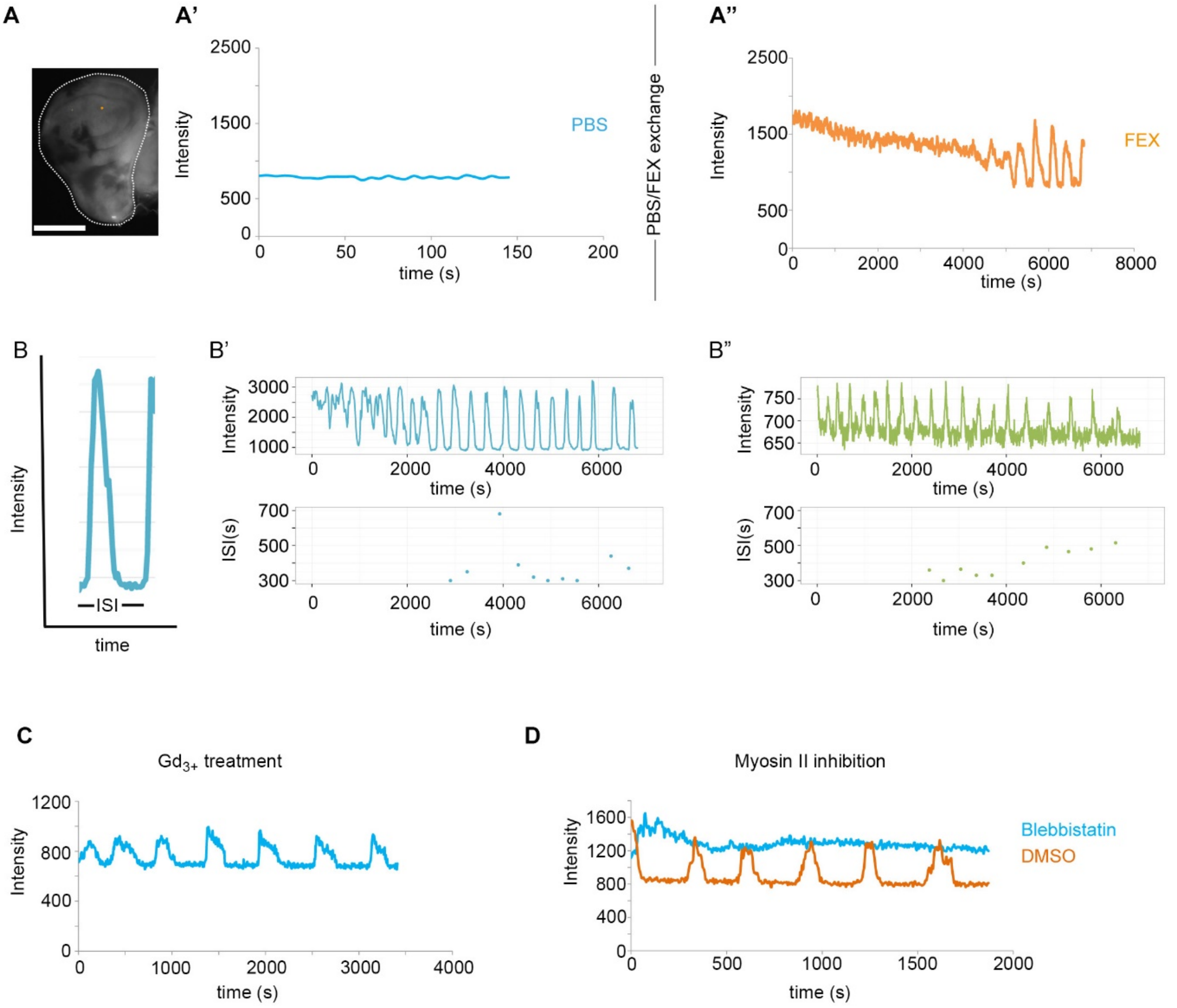

(A-A") Another example of the delay in onset of calcium oscillations. Movie still of one representative wing disc (A) cultured in PBS (A') and subsequently in Schneider's medium supplemented with 5% fly extract (A"). GCaMP-activity was recorded at positions indicated by an orange point (A).

(B-B") Inter-spike interval were quantified as shown in (B) and as described in [6] from intensity tr aces of individual grid points (B',B''). Traces in B' and B'' are examples from the other two discs analyzed in Fig. 4F, G.

(C, D) GCaMP fluorescence intensity traced over time in wing discs cultured in Schneider's medium supplemented with 5% of a fly extract in the presence of 100 µM Gd^{3+} (C) or in the presence of DMSO (orange trace in D) and 100 µM Blebbistatin (blue trace in D).

FEX = fly extract.

Figure S6
A model for calcium oscillations induced by a calcium-mobilizing ligand

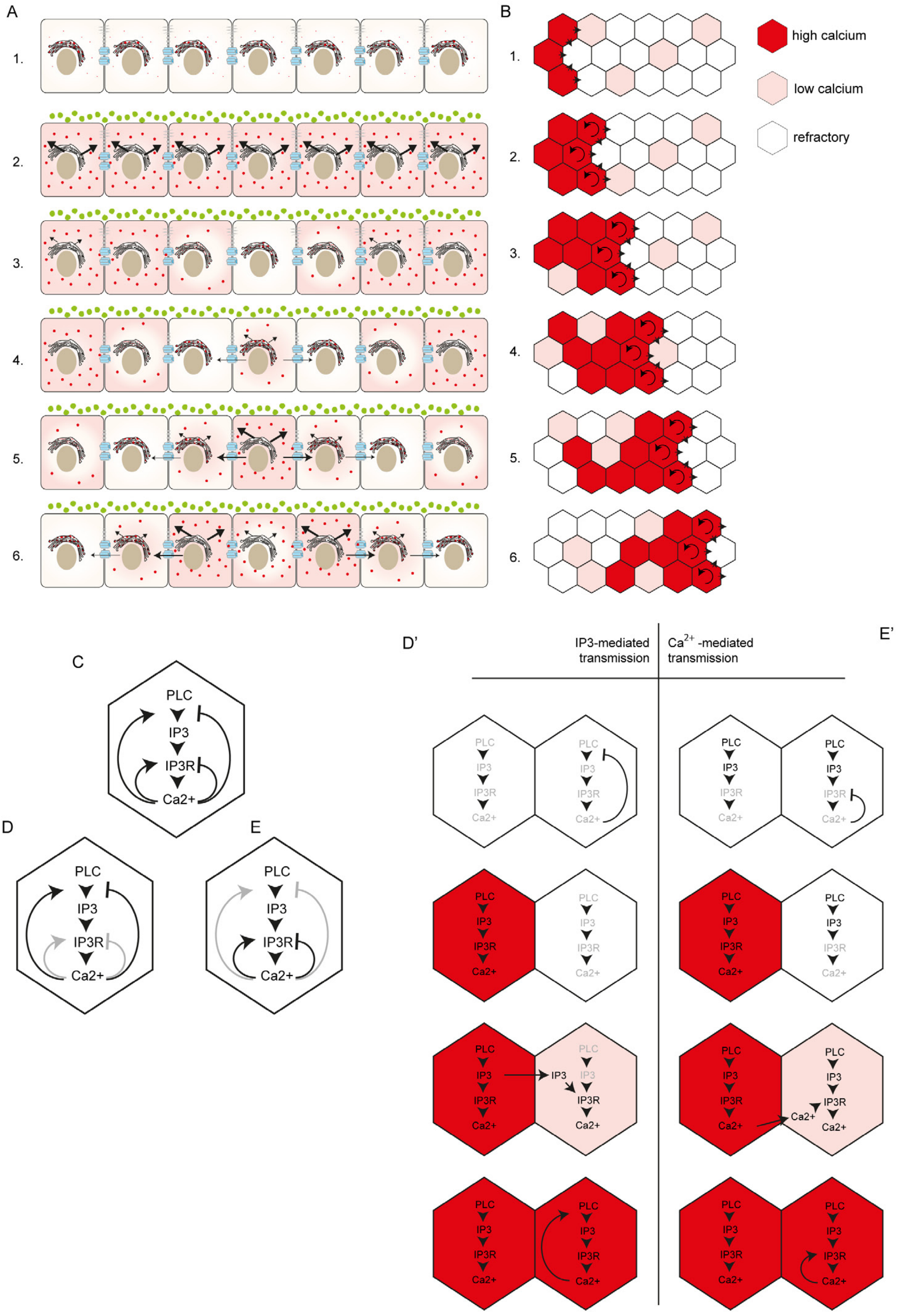

(A) 1. A monolayer of epithelial cells. Refer to Figure S1B for cellular components illustrated. 2. Cytosolic calcium (red) levels increase in all cells when the calcium-mobilizing ligand (green) triggers an IP3-mediated calcium release from ER stores. 3. Calcium is removed from the cytoplasm by re-uptake into the ER in the centrally positioned cells depicted in this image. Within the plane of the tissue, these cells are randomly positioned as a consequence of stochastic heterogeneity in the cell population. As IP3 production and IP3 receptor opening is inhibited by IP3/calcium, cells enter a refractory period during which ligand stimulation or diffusion of IP3/calcium into neighboring cells is not able to induce calcium release (see Fig. S6C-E'). 4. Calcium release from the ER in centrally positioned cells can be initiated again after the refractory period ceases. 5. Ligand-mediated IP3-production and diffusion of IP3/calcium from centrally positioned cells triggers a new wave of calcium release in neighboring cells. 6. Re-uptake of calcium into the ER in the centrally positioned cells followed by steps 3 through 6 gives rise to oscillatory calcium waves.

(B) A field of cells with high cytosolic calcium (red), and early (pink) or late (white) in the period refractory to calcium mobilization. Regenerative amplification (curved arrow) of an inductive signal transmitted from cell to cell (straight arrow) facilitates wave front coordination. Wave front propagation is directed towards cells that have not mobilized calcium for the longest and are therefore more likely to exit the refractory period. Cell heterogeneity causes random fluctuations as to when cells enter refractory period at the trailing wave edge. Fluctuations at the trailing edge suggest that refraction is independent of cell-cell communication.

(C) Previously identified feedback regulation of calcium/IP3 signaling: Calcium activates and inhibits IP3R in a concentration-dependent manner and thereby affects receptor opening probability in response to IP3. Calcium activates and inhibits IP3 production by Phospholipase C (PLC) thereby affecting IP3 availability. IP3 positively influences its own production thereby contributing to signal amplification (not shown, as effect is similar to calcium positively influencing IP3 production). These feedback regulations are discussed in multiple recent reviews, such as [7-9].

(D, D') In a model based on IP3 diffusion between neighboring cells, positive and negative feedback regulation of IP3 production dominate the system. (D') The refractory dynamics are dominated by inhibition of IP3 production in response to the calcium. The whole pathway, including IP3 production, is therefore off, despite the uniform presence of an external calcium-mobilizing ligand. Diffusion of IP3 from neighboring cells promotes IP3R opening and IP3 or calcium-dependent IP3 production.

(E, E') In a model based on calcium diffusion between neighboring cells, positive and negative feedback regulation of IP3R opening probability dominate the system. (E') The refractory dynamics are dominated by inhibition of IP3R channel opening by calcium. The pathway is therefore interrupted at the level of IP3R opening, but not IP3 production. Diffusion of calcium from neighboring cells sensitizes the IP3 receptor to the IP3 pool produced in response to ligand stimulation, thereby opening IP3R and amplifying the signal.

However, a model involving both IP3 and calcium diffusion may account for the entire complexity of wave front propagation dynamics.

Figure S7

Calcium regulates cell survival, tissue architecture and patterning in the wing disc epithelium Related to Figure 5.

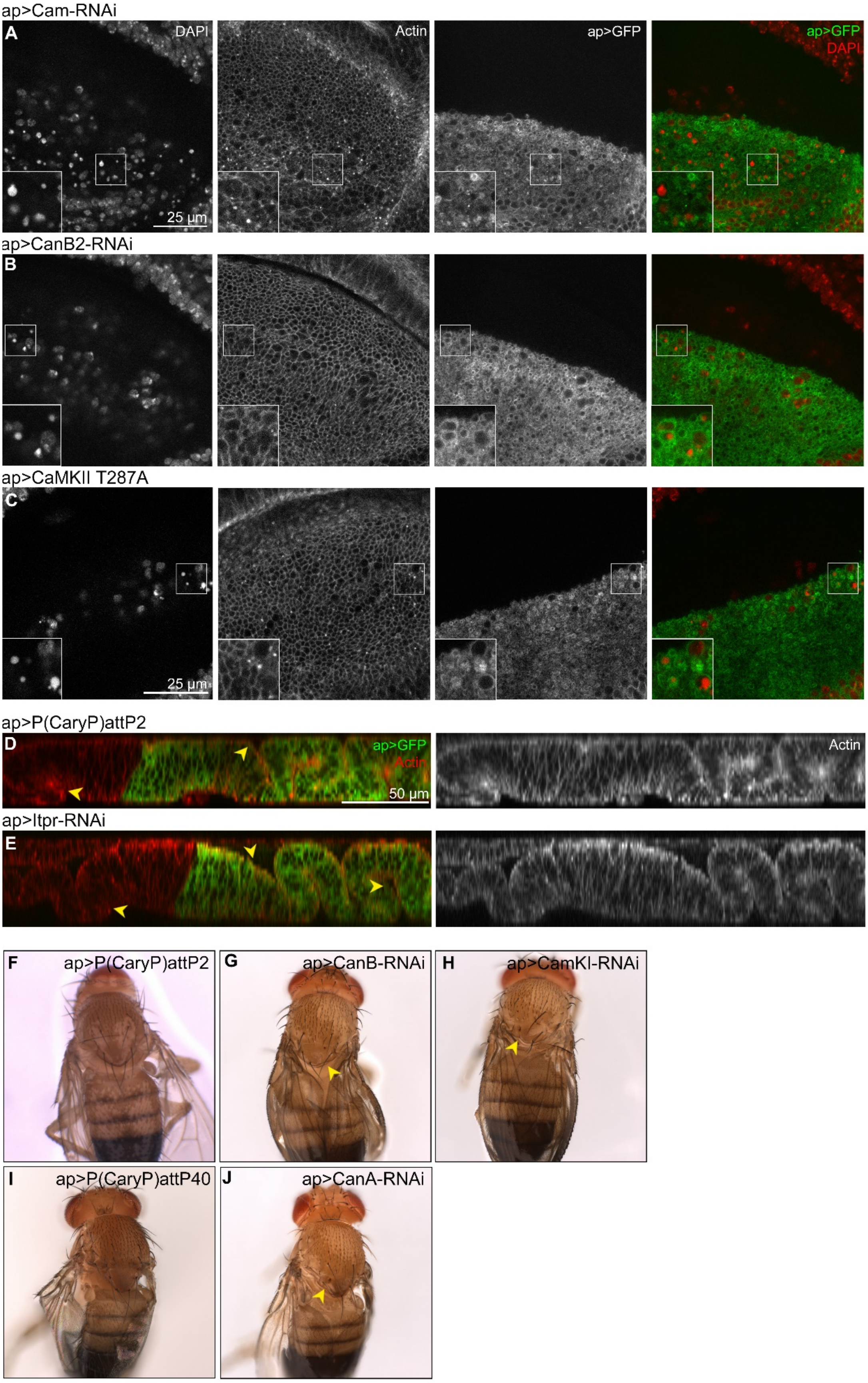

(A-C) Genetic manipulation of calcium signaling causes apoptosis. Confocal XY-sections of wing discs expressing Cam-RNAi (A), CanB2-RNAi (B) and CamKII-T287A (C) under the control of *apGAL4*, which also drives GFP expression (3rd column). Non-GFP expressing cells represent internal wild type control tissue. Discs were stained for DAPI (1st column) and Actin (2nd column). In overlays (4th column) GFP is shown in green, DAPI in red. Note that DAPI-dense vesicles are apoptotic bodies. Scale bars as indicated.
(D, E) Genetic manipulation of calcium signaling alters tissue architecture. Confocal XZ cross-sections of control wing discs (D) or wing discs expressing IP3R-RNAi (E) under the control of the *apGAL4*, which also drives GFP expression (green). Discs were stained for Actin (grey in 2nd column, red in overlay). Arrowheads point to differences in cell height and arrows to differences in dorsal (GFP-positive) and ventral (GFP-negative) tissue fold formation. Scale bar as indicated.
(F-J) Genetic manipulation of calcium signaling alters patterning. Control adult thoraces (F, I) and thoraces expressing CanB-RNAi (G), CamKI-RNAi (H); and CanA-RNAi (J) under the control of *apGAL4*. Arrowheads point to defects in the scutellum structure.

References (Supplement)

1 Lee, M. C. & Spradling, A. C. The progenitor state is maintained by lysine-specific demethylase 1-mediated epigenetic plasticity during Drosophila follicle cell development. *Genes Dev* **28**, 2739-2749, doi:10.1101/gad.252692.114 (2014).

2 Soboloff, J., Rothberg, B. S., Madesh, M. & Gill, D. L. STIM proteins: dynamic calcium signal transducers. *Nat Rev Mol Cell Biol* **13**, 549-565, doi:10.1038/nrm3414 (2012).

3 Clapham, D. E. Calcium signaling. *Cell* **131**, 1047-1058, doi:10.1016/j.cell.2007.11.028 (2007).

4 Chorna, T. & Hasan, G. The genetics of calcium signaling in Drosophila melanogaster. *Biochim Biophys Acta* **1820**, 1269-1282, doi:10.1016/j.bbagen.2011.11.002 (2012).

5 Brown, J. B. *et al.* Diversity and dynamics of the Drosophila transcriptome. *Nature* **512**, 393-399, doi:10.1038/nature12962 (2014).

6 Thurley, K. *et al.* Reliable encoding of stimulus intensities within random sequences of intracellular Ca2+ spikes. *Sci Signal* **7**, ra59, doi:10.1126/scisignal.2005237 (2014).

7 Dupont, G., Combettes, L., Bird, G. S. & Putney, J. W. Calcium oscillations. *Cold Spring Harbor perspectives in biology* **3**, doi:10.1101/cshperspect.a004226 (2011).

8 Leybaert, L. & Sanderson, M. J. Intercellular Ca(2+) waves: mechanisms and function. *Physiological reviews* **92**, 1359-1392, doi:10.1152/physrev.00029.2011 (2012).

9 Konieczny, V., Keebler, M. V. & Taylor, C. W. Spatial organization of intracellular Ca2+ signals. *Semin Cell Dev Biol* **23**, 172-180, doi:10.1016/j.semcdb.2011.09.006 (2012).
